# Non-cellular immunotherapies in pediatric central nervous system tumors

**DOI:** 10.3389/fimmu.2023.1242911

**Published:** 2023-10-11

**Authors:** Sarah Rumler

**Affiliations:** Department of Pediatrics, Medical College of Wisconsin, Milwaukee, WI, United States

**Keywords:** pediatric oncology, neuro-oncology, pediatric neuro-oncology, immune-oncology, immune checkpoint inhibitors, cancer vaccines, radiation therapy, brain tumors

## Abstract

Central nervous system (CNS) tumors are the second most common type of cancer and the most common cause of cancer death in pediatric patients. New therapies are desperately needed for some of the most malignant of all cancers. Immunotherapy has emerged in the past two decades as an additional avenue to augment/replace traditional therapies (such as chemotherapy, surgery, and radiation therapy). This article first discusses the unique nature of the pediatric CNS immune system and how it interacts with the systemic immune system. It then goes on to review three important and widely studied types of immune therapies: checkpoint inhibitors, vaccines, and radiation therapy, and touches on early studies of antibody-mediated immunogenic therapies, Finally, the article discusses the importance of combination immunotherapy for pediatric CNS tumors, and addresses the neurologic toxicities associated with immunotherapies.

## 1 Introduction

Central nervous system (CNS) lesions are a common cause of childhood cancer. In the United States in 2023, CNS tumors were second only to hematologic, myeloproliferative, and myelodysplastic malignancies in terms of prevalence (26% vs 28%, respectively) (1). Prognosis for pediatric CNS tumors varies widely: some high-grade gliomas are universally fatal, and some low-grade gliomas have survival rates approaching 100%. While pediatric mortality due to CNS tumors has decreased over the past 40 years, there has been no significant change in brain and other CNS tumor mortality in children and adolescents since 2007. Therefore, CNS tumors are the leading cause of cancer-related death in pediatric patients (2). New, efficacious, and more durable therapies are desperately needed for pediatric CNS tumors. Immunotherapy has emerged as an additional component in cancer treatment (including surgery, radiation, and traditional chemotherapy). Immune therapy utilizes the patient’s immune system to augment the already inherent anti-tumor capabilities, as monotherapy or in combination with other therapies. The goal of this review is to first introduce the unique nature of the pediatric CNS immune system and review current and future progress in select immunotherapies currently available to treat pediatric patients.

## 2 The immunologic landscape

### 2.1 The immune environment of the CNS

The human CNS is a tightly-regulated, well-evolved system. Previously thought to be tightly sealed away from the rest of the body (the idea of “immune privilege”), as we have learned in the last 30-40 years, the CNS *does* interact with the systemic immune system (3–6) in its own unique and regulated way, feasibly because immune-mediated inflammation of the brain could be extremely detrimental to an organism (7, 8). The blood-brain barrier (BBB) is a unique feature of the CNS microvasculature anatomy that tightly regulates the influx and efflux of molecules, substances, fluids, and cells, including immune cells (9). The interaction between the overall immune system (10) and the CNS system includes specific structures that allow this interaction (3). Specialized pathways deep in the dura (the so-called glymphatic system) (3–5, 11) direct interstitial fluid from the CNS towards drainage into the deep cervical lymph nodes and allow interaction with the systemic immune network (10, 12, 13). The pediatric BBB and immune environment is unique when compared to the adult. The junctions of the pediatric BBB are tighter and are known to be developed by the time an infant is born (14). Additionally, despite being immunologically naïve, the young infant CNS is entirely capable of mounting a robust immune response (15), In contrast, age-related stresses and inflammation affect the function and structure of the aged CNS. Additionally, older adults have stiffer, more permeable vasculature in general (15, 16). Furthermore, the expression of transmembrane transport proteins varies in pediatric tissue studies when compared to adult (17). This can affect the influx and efflux of substances into and out of the CNS, including proteins, drugs, and other blood-borne substances (14, 16, 17).

### 2.2 Tumor microenvironment and the blood-brain-tumor barrier

To understand how CNS lesions respond to immunotherapies, the CNS tumor microenvironment (TME) must be considered in the context of the known differences of the pediatric BBB and tumor microvasculature (the blood-brain-tumor barrier, BBTB), especially due to its unique qualities. TME includes the collection of cells (particularly immune cells and other blood cells), blood vessels, stromal cells, and extracellular matrix surrounding a tumor (18–20). In consideration of the immune landscape of tumors, there are “hot” and “cold” tumors. “Hot” tumors are more immunologically active, more prone to inflammatory responses, and have a higher number of active tumor-infiltrating lymphocytes (TILs). In contrast, “cold” tumors have TILs that lower in number and have an exhausted phenotype (6, 20–22). The resident macrophages of the brain are microglia. These cells tightly regulate the immune homeostasis of the CNS and are known to have powerful immunosuppressive functions (21–26). Tumor-associated macrophages (TAMs) are a well-known part of the TME. In brain tumors, TAMs are bone-marrow derived (whereas microglia are resident in normal brain) (27, 28). TAMs are thought to “polarize” depending on signaling, stimulus, and pathology into either pro-inflammatory (immunoactive) or anti-inflammatory (immunosuppressive) states (27–30). Brain tumor-associated TAMs are pro-tumorigenic in a number of ways, such as upregulation of endothelial cell secretion of vascular endothelial growth factor (VEGF), secretion of immunosuppressive cytokines such as transforming growth factor beta (TGF-β) and interleukin 10 (IL-10), secretion of arginase (to starve T-cells), secretion of epidermal growth factor (EGF) to promote tumor migration, secretion of pro-tumor chemokines and cytokines, secretion of prostaglandins (to inhibit activation of T-cells), and direct antigen presentation functions (30–33). Additional non-TAM myeloid-derived suppressor cells (MDSCs), are a group of cells of myeloid origin that are pathologically activated and serve an immunosuppressive function against both adaptive and innate anti-tumor immunity (34–36).

**Figure 1:**
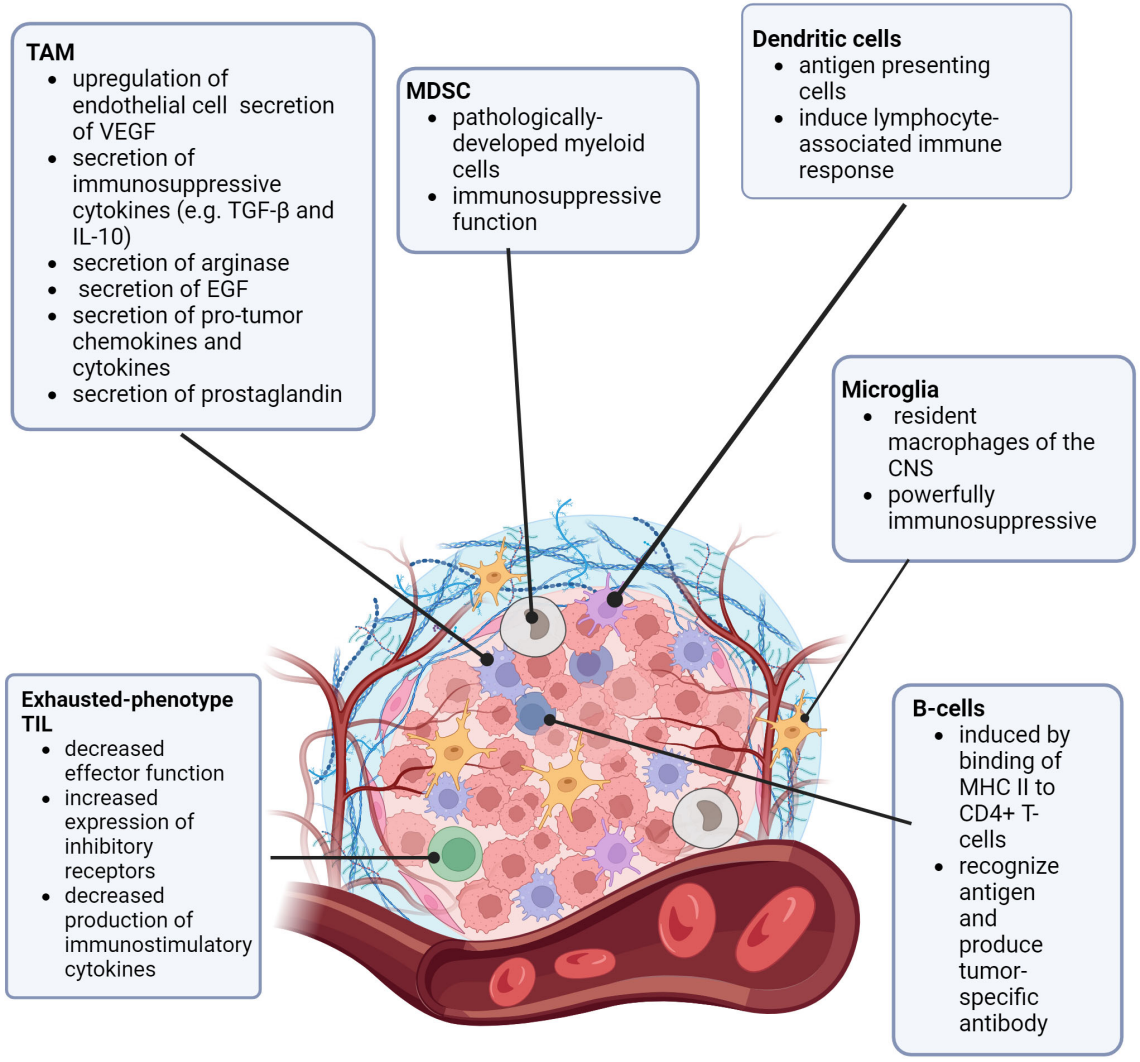
The Pediatric CNS Tumor Microenvironment. TAM (tumor associated macrophage), MDSC (myeloid derived suppressor cell), VEGF (vascular endothelial growth factor), TGF-β (transforming growth factor β), IL-10 (interleukin 10), EGF (epidermal growth factor), MDSC (myeloid-derived suppressor cell), CNS (central nervous system) MHC (major histocompatibility complex), CD4 (cluster of differentiation 4), TIL (tumor-infiltrating lymphocyte).

While most of the research on the CNS TME is in adult gliomas (37–39), more information is becoming available regarding the pediatric CNS TME, and how it relates to the pediatric microvasculature and BBB permeability (26). In general, pediatric CNS tumors are considered “cold”, due to the presumed lack of genetic mutations (generation of neoantigens) (21, 40). Additionally, pediatric tumors tend to have loss of expression of major histocompatibility complex I (MHC I), which decreases the ability of T-lymphocytes to recognize and become activated (41). In contrast to adult CNS tumors, the pediatric CNS TME has a relatively high concentration of immunosuppressive cells, such as marrow-derived TAMs, resident microglia, and other MDSCs (27, 41, 42). **Figure 1** is a review of the pediatric CNS-specific TME. As mentioned before, the TILs that are present in pediatric CNS tumors exhibit an exhausted phenotype. These TILs have decreased effector function, increased expression of inhibitory receptors, and decreased production of immunostimulatory cytokines (42), further decreasing the recruitment of additional immune response. Notably, there is some variation in the TME of different types of pediatric CNS lesions (21, 39–46). Griesinger, et al. (47) used flow cytometry to analyze the phenotype and frequency of immune cells that infiltrate tumors in the most common pediatric brain tumor types (pilocytic astrocytoma, ependymoma, glioblastoma, and medulloblastoma). Pilocytic astrocytoma and ependymoma demonstrated a significantly higher number of infiltrating myeloid and lymphoid cells, more of which were activated compared with glioblastoma, medulloblastoma, or nontumor tissue. Levine, et al. (48) designed a 103-gene immune-oncology transcriptomic panel elucidating immune cell types and tumor inflammatory signatures (TIS, a biomarker for immune response to immune checkpoint inhibitors). Pediatric glioneuronal tumors had substantial upregulation of T-cell markers and regulatory genes; diffuse astrocytomas had a nearly normal profile. Diffuse intrinsic pontine gliomas (DIPGs) showed strong upregulation of macrophage markers. In work done by Wei, et al. (49), patient-derived xenograft (PDX) models of pediatric high-grade gliomas (pHGG) and diffuse midline gliomas (DMG) were used to examine the vascular permeability and molecular transport differences between these two types of lesions. Staining for specific tumor cell markers hVimentin and endothelial marker CD31 revealed minimal change in the vasculature of DMGs, but significant alterations in vasculature (such as an increased number of branch points and overall vasculature density) in pHGG. Additionally, when staining for tight junction marker Claudin5 (Cldn5, a marker for tight junctions) and Glut1 (a BBB-associated glucose transporter, also known as Slca2a1), differences were noted in the expression of these proteins in the vasculature of pHGG PDX models when compared to normal brain and DMG PDX models. pHGG models showed slightly decreased expression of Glut1 and increased and more disorganized expression of Cldn5, while the DMG vasculature was far similar to the expression patterns of these proteins in normal brain vasculature. In a review by Morris, et al. (50), similar differences in expression patterns were noted across different subtypes of medulloblastoma including wingless (WNT), sonic hedgehog (SHH), and group 3. The heterogeneity of the TME, BBB, and BBTB when compared to pediatric versus adult tumors, and the variability across different pediatric tumor types, could help to explain why some CNS tumors respond better than others to various types of immune therapies, both in the pediatric arena and when compared to adult tumors (40–46).

## 3 Immunotherapies for pediatric CNS disease

There are multiple types of non-cellular immunotherapies available for pediatric CNS disease and some interesting therapies are on the horizon. Here, we review a selection of promising immune-oncologic therapies available and the research data available. Of note, this article considers the general category of antibody therapy as targeted therapy, as opposed to immune therapy, and will discuss antibody therapies in the context of immunogenicity.

### 3.1 Checkpoint inhibitors

Part of the standard immune regulation and surveillance in humans is by regulation and modulation of the innate immune system. One way this is accomplished is by immune checkpoint regulation. When programmed cell death 1 (PD1, which is found on the surface of T- lymphocytes, among other immune cells) binds with programmed cell death ligand 1 (PDL-1, found on the surface of most mammalian cells) it dampens the immune response and makes the T-cell mediated kill less effective (35). Similarly, T-lymphocyte-associated protein 4 (CTLA4, expressed on the surface of activated T-lymphocytes) binds to CD80/CD86 (found on antigen-presenting cells), which sends an inhibitory signal to the activated T-cell, as another checkpoint mechanism (51–54). Many types of tumor cells have evolved ways to evade immune detection or cell-kill mechanisms (such as over-expression of PDL1). Immune checkpoint inhibitors (ICIs) are monoclonal antibodies that bind to either PDL1, PD1, or CTLA4 to overcome this dampening down of the immune response, leading to better tumor cell kill by increased immune infiltration (54).

Multiple iterations of immune checkpoint inhibitors are approved for many types of cancers, including cancers of the breast, skin, lung, bladder, colon/rectum, esophagus, cervix, kidney, uterus, liver, some sarcomas, and some lymphomas. Unfortunately, the efficacy of checkpoint inhibitors alone in CNS tumors has been lackluster in both adult and pediatric populations (54–61). CheckMate-908, a study using nivolumab+ ipilimumab in pediatric patients with high-grade CNS malignancies showed similar results, with failure to demonstrate clinical benefit (62).

The lack of efficacy of ICIs to treat most CNS malignancies is likely multifactorial. Firstly, as detailed above, many lesions of the CNS are known to be “cold” in terms of immunogenicity. One of the major barriers to effective ICI action in most CNS tumors is the fact that ICIs rely on the activation of lymphocytes to exert their mechanism of action (63–65). If the T-cells are activated in the periphery, and only a minimal amount enters the CNS, the point is moot (65). A second possible reason is the challenge of larger molecules to pass through the BBB (however, there is evidence to suggest that ICI are effective in treating metastatic lesions to the brain, which suggests that the BBB is at least partially permeable to ICI therapy.) (66, 67) The lack of efficacy in pediatric patients could also relate to the relatively less permeable state of the pediatric BBB when compared to the aged one (14–17, 46). As the use of ICIs in pediatric patients as monotherapy has not shown clinical efficacy for most CNS tumors (45, 62, 68), many of the trials currently open using ICIs for pediatric patients also include other treatment modalities, such as chemotherapy, radiation therapy, multiple ICIs, or vaccinations.

Notably, there is one population where monotherapy with ICIs has shown efficacy, and this is patients with constitutional mismatch repair deficiency (CMMRD). This is an autosomal recessive condition characterized by mutations in DNA repair genes, which tends to result in the nearly universal development of malignancies (69). Because these patients have such a high tumor mutational burden, there are multiple case reports and emerging data that these patients in particular could benefit from monotherapy with ICIs (70). **Table 1** outlines the current open clinical trials in pediatric patients involving ICIs, +/- other chemotherapies.

**Table 1:** Current open clinical trials for pediatric patients using immune checkpoint inhibitors +/- other chemotherapies.

Clinical Trial Identifier	Drug	Population	Phase of Study	Status
NCT02793466	Durvalumab	Patients >12 months-<18 years of age with relapsed or refractory solid tumors (including CNS)	I	Active, not recruiting
NCT04416568	Nivolumab + ipilimumab	Patients >6 months-<40 years of age with relapsed or refractory malignant rhabdoid tumor, rhabdoid tumor of the kidney, epitheloid sarcoma, chordoma, other INI1-negative or SMARCA4-deficient malignant tumors, atypical teratoid rhabdoid tumor (ATRT), other INI1-negative or SMARCA4-deficient primary CNS malignant tumors	II	Active, recruiting
NCT04323046	Ipilimumab + nivolumab	Patients aged 6 months-22 years with recurrent or progressive high-grade glioma (HGG)	I	Active, not recruiting
NCT05468359	Atezolizumab + sorafenib, bevacizumab, and cyclophosphamide	Patients < 30 years of age with refractory or recurrent solid tumors	I/II	Active, recruiting
NCT05407441	Nivolumab + ipilimumab, with tazemetostat	Patients ages 6 months-21 years with ATRT, other INI1- or SMARCA4-deficient primary CNS malignant tumors, malignant rhabdoid tumor, rhabdoid tumor of the kidney, epitheloid sarcoma, chordoma, other INI1-negative or SMARCA4-deficient malignant tumors	I/II	Active, recruiting
NCT05465174	Nivolumab with tovorafenib	Patients ages 1-39 years with newly diagnosed or recurrent craniopharyngioma	II	Active, recruiting
NCT02359565	Pembrolizumab	Patients aged 1-18 (up to 22 in efficacy portion) years with recurrent, progressive, or refractory non-brainstem HGG, ependymoma, medulloblastoma	I	Active, recruiting
NCT05081180	Avelumab with Lenvatinib	Patients aged 2-18 years with progressive high-grade histology CNS malignancy, patients with diffuse midline glioma +/- H3K27M mutation that has not progressed	I	Active, recruiting
NCT03838042	Nivolumab, entinostat	Patients aged 6-21 years with CNS tumors: medulloblastoma, ependymoma, ATRT, ETMR, pediatric high-grade glioma (including DIPG), or other pediatric embryonal CNS tumors OR solid tumors: neuroblastoma, nephroblastoma, rhabdoid tumor, embryonal or alveolar rhabdomyosarcoma, other embryonal small round blue cell tumors including pediatric type (bone) sarcoma or other pediatric type solid tumors OR Children and adolescents with newly diagnosed high-grade glioma (HGG) in the context of a constitutional mismatch repair deficiency	I/II	Active, recruiting

### 3.2 Anti-tumor vaccines

**Figure 2:**
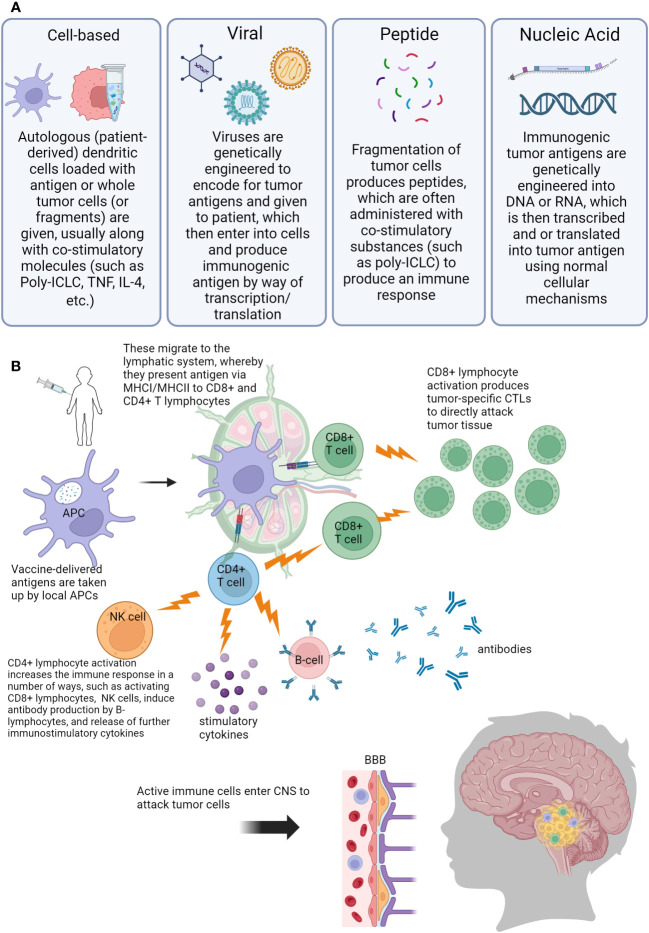
Visual overview of concepts of anti-tumor vaccination for pediatric central nervous system (CNS) tumors. **(A)** Description of different vaccine types used **(B)** Overview of immune response generated by vaccination. Abbreviations: Poly-ILCL (polyinosinic-polycytidylic acid stabilized with carboxymethylcellulose and poly-L-lysine), TNF (tumor necrosis factor), IL-4 (interleukin 4), DNA (deoxyribonucleic acid), RNA (ribonucleic acid), APC (antigen presenting cell), MHC (major histocompatibility complex), CD4/CD8 (cluster of differentiation 4/8), NK (natural killer), CTL (cytotoxic T-lymphocyte), BBB (blood-brain barrier), CNS (central nervous system).

As more targeted approaches are always needed, the development of anti-tumor vaccines is an exciting prospect in harnessing the immune system to control CNS tumors in a controlled and specific way. Cancer vaccines have been in development since the 1980s when autologous tumor cells were administered to patients with colorectal cancer (71, 72). Currently, there is renewed interest in utilizing cancer vaccines, especially for pediatric patients. This is in part due to the wide availability of laboratory capabilities to identify appropriate tumor antigens, in addition to our growing knowledge about the behavior of dendritic cells (DCs), which are a vital factor for vaccination efficacy (71–73). DCs are professional antigen-presenting cells (APC)with a powerful ability to stimulate the immune system. DCs can travel to lymph nodes, and process and cross-present antigens via MHC I and MHC II. When naïve CD8+ T-lymphocytes are presented with tumor antigen, this then causes differentiation into tumor-specific cytotoxic T-lymphocytes (CTLs) (74). DCs also interact with CD4+ T-lymphocytes via MHC II, which provides help for CD8+ CTLs via the release of cytokines to boost the immune response (74, 75). Additionally, through crosstalk mechanisms, DCs stimulate and activate natural killer (NK) cells, which are another vital arm of the anti-tumor immune response (75–79). Multiple different types of vaccines have been developed to treat tumor cells: cell-based, virus-based, nucleic acid-based, and peptide-based vaccines (80). **Figure 2** is a visual review of the vaccines covered in the next paragraphs, that are the most widely studied in pediatric CNS malignancies. **Table 2** outlines current open clinical trials for pediatric patients using vaccines, with and without other chemotherapies or procedures.

**Table 2:** Current open clinical trials for pediatric patients using vaccines, +/- other chemotherapies or procedures.

Clinical Trial Identifier	Vaccine/Target	Population	Phase of Study	Status
NCT03988283	Personalized neoantigen DNA vaccine	Any patient between the ages of 12 and 39 years of age (inclusive) who was diagnosed with a pediatric brain tumor of any histologic subtype, who has now developed recurrent or refractory disease, and who already received standard-of-care therapy.	I	Not yet recruiting
NCT03299309	Novel pp65 peptide vaccine (PEP-CMV), temozolomide	Patients aged 3-35 years with recurrent/progressive medulloblastoma and malignant glioma	I	Active, not recruiting
NCT03043391	PVSRIPO	Patients aged 12-21 years with recurrent supratentorial WHO Grade III malignant glioma (anaplastic astrocytoma, anaplastic oligoastrocytoma, anaplastic oligodendroglioma, anaplastic pleomorphic xanthoastrocytoma, ependymoma) or WHO Grade IV malignant glioma, medulloblastoma, or atypical teratoid/rhabdoid tumor (ATRT)	I	Active, not recruiting
NCT04978727	SurVaxM	Patients aged 1-21 years with histologically confirmed diagnosis recurrent or progressive Medulloblastoma, Glioblastoma multiforme (GBM), Anaplastic astrocytoma, High-grade astrocytoma, NOS, Anaplastic oligodendroglioma, Anaplastic ependymoma (WHO Grade III), Ependymoma (WHO Grade II), or newly diagnosed radiologically confirmed DIPG	I	Active, recruiting
NCT04837547	TTRNA DC vaccine, TTRNA-x-ALT, Autologous HSC	Patients aged 1-30 years with recurrent neuroblastoma or newly diagnosed DIPG patients who are at least 3 years of age	I	Active, recruiting
NCT04749641	H3.3-K27M Neoantigen Vaccine	Patients aged 5 years and older with newly diagnosed DIPG	I	Active, recruiting

#### 3.2.1 Cell-based vaccines

In addition to their important role in the vaccine-generated immune response, the most frequently used type of cell-based vaccination is made of DCs loaded with some type of tumor antigen (such as tumor lysate) (80–82). DC vaccines are typically made from a patient’s own autologous DCs and often have to be cultured in the presence of cytokines and growth factors (such as GM-CSF, IL-4, and TNF) for full differentiation and activation. After they are expanded, they can be given to the patient with other substances, such as polyinosinic-polycytidylic acid (poly I:C) or poly I:C stabilized with carboxymethylcellulose and poly-L-lysine (poly-ICLC), which serve as co-stimulatory compounds to help increase the immune response of the vaccine (80–83). Other cell-based vaccines include whole tumor cell or cell fragment vaccines (often generated by human tumor cell lines), which can generate a robust immune response due to a higher number of antigens present on whole cells or cell fragments. Similarly to DC vaccines, these cell-based vaccines are often engineered to produce various cytokines or stimulatory molecules to increase the immune response (80, 84, 85). There have been multiple studies in the past twenty years looking at the effect of DC vaccines in pediatric CNS tumors. In one study by Ardon et al. (86), forty-five children with relapsed malignant brain tumors (HGG, medulloblastoma (MB)/primitive neuro-ectodermal tumor (PNET), atypical teratoid rhabdoid tumor (ATRT)) showed promising responses to DCs loaded with patient tumor lysates. Median overall survival (OS) for HGG, glioblastoma multiforme, and anaplastic astrocytoma was 13.5, 12.2, and 18.4 months, respectively. The authors noted the most favorable responses in patients with HGG and ATRT compared to other malignant CNS tumor types. Another study by Rutkowaski, et all (87) for twelve children and adults with recurrent glioma reviewed the extent of resection and outcomes with DCs loaded with patient tumor homogenate. In patients with residual disease, 1 of 6 had a partial response, and 1 of 6 had stable disease. In patients whose tumors were completely resected, 2 of 6 had complete clinical responses for at least 3 years post-study. A pilot study by Olin, et al. (88) had 12 pediatric and adult patients (8 of whom received the DC vaccine) with recurrent primary brain tumors given GBM6-AD/DC vaccine. Unfortunately, the best response observed was a partial response in 1 patient, however, the vaccine was found to be well-tolerated and forms a basis for future studies.

#### 3.2.2 Viral vaccines

Viral delivery systems are an obvious choice for vaccine development. Viruses are naturally immunogenic and enter and modify cells as the natural mechanism of propagation (89). Additionally, the use of viral vectors has increased greatly due to our knowledge of manipulating viruses to use as treatment vectors. Viruses used in the development of anticancer vaccines are chosen for several reasons, such as high immunogenicity (adenovirus, poxvirus, alphavirus) (89–92) or oncolytic nature (measles virus, herpes simplex virus, vesicular stomatitis virus) (89). Viruses can be extensively engineered and are simpler and cheaper to produce and store (80). Based on promising adult data, Gállego Pérez-Larraya et al. (93) published a study for pediatric patients with newly diagnosed DIPG. Twelve patients received dose-escalating intratumoral injections of DNX-2401, an oncolytic adenovirus, and 11 of these patients then received subsequent radiotherapy. Nine patients experienced a reduction in tumor size, with median survival was 17.8 months. There was one patient who was still alive at 38 months. Friedman (94), et al. conducted a study that included 12 pediatric patients with recurrent or progressive supratentorial high-grade gliomas who were given intratumoral G207 (oncolytic HSV-1 viral) injections, with or without a single dose of 5 Gy radiation. 11 patients showed radiographic, neuropathological, or clinical responses. Median OS was 12.2 months, and 4 out of 11 patients survived at least 18 months. Schuelke, et al. (95) published results of a trial that enrolled 6 pediatric patients with high-grade brain tumors who received 3 days of subcutaneous sargramostim followed by 2 days of intravenous pelareorep (an immunomodulatory oncolytic reovirus). All patients progressed on therapy after a median of 32.5 days and died a median of 108 days after recruitment (96).

#### 3.2.3 Peptide vaccines

Peptide vaccines are vaccines engineered from fragments of tumor cell material and often have multiple tumor epitopes (10–20) included in their repertoire. They are also often administered with co-stimulatory substances to help with the generation of immune responses. They are designed to generate an *in vivo* endogenous immune response in a patient, by way of APC-MHC-T lymphocytes as described earlier. Peptide vaccines are synthetic, cheaper, and less laborious to produce when compared to DC vaccines, for example (80, 84). One breakthrough in peptide vaccinations is the development of K27M-directed therapy. H3.3K27M mutations are features of extremely aggressive high-grade midline gliomas. These lesions are nearly universally fatal. Ochs, et al. (97) demonstrated the targetability of H3K27M as a specific epitope with a peptide vaccine that induced promising immune responses in murine models. In the clinical space, Mueller, et al. (98) described the results from a trial including newly diagnosed patients with DIPG (stratum A) or non-pontine DMG (stratum B) aged 3-21 years who were treated with an H3.3K27M-targeted peptide vaccine, which was administered in combination with poly-ICLC every 3 weeks for 8 cycles, followed by once every 6 weeks. A total of 29 patients (19 in stratum A and 10 in stratum B) enrolled in the study. OS at 12 months was 40% (stratum A) and 39% (stratum B). Perhaps not surprisingly, the median OS was higher for patients who had an expansion of H3.3K27M-reactive CD8+ T cells compared to those who did not (16.1 months and 9.8 months, respectively). Pollack, et al. (99–101) published a series of three separate pilot clinical trials using peptide vaccines for pediatric patients with gliomas. All three followed a similar vaccine schedule (intramuscular injections given every 3 weeks for eight courses, followed by booster vaccinations every 6 weeks), and used a vaccine composed of an emulsification of a panel of three common glioma-associated antigens (IL-13Ra2, EphA2, and survivin) administered with Poly-ICLC. All three studies demonstrated the safety and positive immunogenicity of the vaccine (main endpoints). The 2014 pilot study enrolled 26 pediatric patients with newly diagnosed brain stem gliomas or other high-grade gliomas, 14 with newly diagnosed brain stem gliomas treated with irradiation 12 with newly diagnosed brain stem glioma or other HGG treated with irradiation and concurrent chemotherapy. Median survival was 13.3 months from diagnosis. Of note, 5 patients in the study experienced pseudoprogression (increase in tumor size or increase in contrast enhancement on MRI). Of these 5 patients, 4 survived ≥ 18 months after diagnosis, compared to 2 of 15 children who did not experience pseudoprogression. Pseudoprogression is often thought to be indicative of increased inflammation of a brain lesion, which could be suggestive of a more robust immune response in these patients (99). Another study by the same group enrolled twelve pediatric patients with recurrent malignant gliomas. Median progression-free survival (PFS) from the start of vaccination was 4.1 months and the median OS was 12.9 months. Six-month PFS and OS were 33% and 73%, respectively. In this study, one patient experienced pseudoprogression and was taken off the study, but had a response on subsequent therapy. Another child had a sustained (≥ 39-month) partial response (100). A third study done by the same group was for pediatric patients with recurrent low-grade gliomas who had received at least 2 prior treatment regimens. Fourteen patients enrolled in the study. One child who experienced pseudoprogression had significant durable regression of tumor (>75%) with regression of metastatic disease, which lasted for >57 months. Three others had sustained PRs (partial responses) (>10, >31, and >45 months) (101).

#### 3.2.4 Nucleic acid–based vaccines

Nucleic acids (NAs) (i.e. deoxyribonucleic acid (DNA) and ribonucleic acid (RNA), are increasingly being used in vaccination development. They can be manufactured quickly, are easy to modify, and can be engineered to express co-stimulatory substances to enhance both humoral (B-cell/antibody driven) and cellular (T-cell driven) immune responses (80). DNA-based vaccines are produced by inserting DNA into a bacterial plasmid to express tumor antigens. RNA-based vaccines are generally composed of a cap, multiple untranslated regions, the region coding for the antigen, and a tail (102). NA vaccines have flexibility in terms of administration, with intramuscular, intradermal, transdermal, mucosal, and intravenous methods of delivery. When DNA vaccines are administered, the cells where they are administered (myocytes, for example) take in the DNA and then express the tumor antigen using their cell machinery. RNA vaccines have fewer “steps” and are closer to translation directly to immunogenic proteins (102, 103). There are many different delivery methods (including physical and chemical) that are exploited to get the NA into the cell nucleus, including nanocarriers, lipid-based systems, electroporation, a gene gun, ultrasound, and laser (71, 79, 84, 101, 103–105). Regardless of the delivery, NA vaccines are another way to generate an *in vivo* immune response. The use of NA vaccines has preclinical evidence but has not been used often in pediatric CNS lesions. There are multiple clinical trials currently open that use NA as an antigen, either on its own or loaded onto DCs. Additionally, DNA-based vaccines advance personalized medicine by being specifically manufactured from a patient’s own tumor DNA on a case-by-case basis (80, 102, 105, 106). There is an abundance of pre-clinical data showing the efficacy of NA vaccines in generating an immune response, however, the use of NA vaccines in pediatric patients is still in the infancy stages, and an exciting prospect in cancer vaccine technology (102–107).

### 3.3 Other antibody-based therapies

There is preclinical data for other types of immunotherapies that perhaps have a future role in the treatment of pediatric CNS malignancies (108, 109). Bispecific T-cell engagers (BiTEs) are molecules that “usher” T-lymphocytes to tumors by having two binding sites (110): one for a T cell and one for a tumor antigen. Bhojnagarwala (111), et al, designed multiple BiTEs targeting IL-13Rα2, which is a glioblastoma (GBM) surface antigen, and tested them in an orthoptic model. One of these molecules, PB01-forward, controlled tumor growth and resulted in longer animal survival. Sun, et al. (112) designed a bispecific antibody (BsAB) with epidermal growth factor receptor variant III (EGFRvIII, an antigen presents on GBM tumor cells) and CD3 as the targets, which demonstrated significant *in vivo* and *in vitro* activity in mouse models (113, 114). CD47 is an anti-phagocytic molecule that is expressed by brain cancer cells. Abbas, et al. (115) treated orthoptic xenograft mouse models of medulloblastoma with either craniospinal irradiation (CSI), anti-CD47 antibody, or both. The group that received both modalities showed marked regression of the tumor. Gholamin, et al. (116) tested a humanized anti-CD47 antibody, Hu5F9-G4, on five types of aggressive pediatric brain tumors (group 3 medulloblastoma (primary and metastatic), ATRT, PNET, pediatric glioblastoma, and DIPG) in patient-derived orthotopic xenograft models, and efficacy was demonstrated in both *in vivo* and *in vitro*.

## 4 Radiation therapy

The effect of radiation therapy (RT) on the care of cancer, in particular brain tumors, cannot be understated. Radiation therapy is included in the standard repertoire of care for most high-grade pediatric brain tumors, including medulloblastoma, high-grade glioma, midline glioma, ependymoma, and high-grade embryonal lesions. Radiation has a myriad of effects on the cancer cell, and emerging research makes the invaluable contribution of radiation therapy on immune-mediated control evident.

### 4.1 Immunomodulatory effects of radiation therapy

It is well-establish that radiation therapy can kill tumors due to directly causing cell death. But it is also known that radiation increases the immunogenicity of a tumor in several ways. Firstly, radiation-induced DNA damage causes DNA to accumulate in the cytoplasm of cells, resulting in danger-associated molecular patterns (DAMPs) (117, 118). After this DNA is recognized as a DAMP by host receptors—in tumors, cyclic guanosine monophosphate (GMP)–adenosine monophosphate (AMP) synthase (cGAS; also known as MB21D1) is thought to be the main mechanism of this process (119). Binding of cGAS to cytoplasmic DNA activates the cGAS, and triggers a cascade in the cell, ultimately resulting in increased expression and release of interferons and cytokines (119, 120). This sterile inflammation acts as a recruitment signal to the immune system, causing infiltration and maturation of dendritic cells (121) with subsequent antigen presentation, thus resulting in activation and tumor infiltration of tumor-specific T-cells (122, 123).

### 4.2 Abscopal effect

In addition to local immune infiltration, RT causes an anti-tumor effect distant from the original radiated field, known as the abscopal effect. First described by Mole in 1953, this phenomenon is somewhat rare and thought to be mediated by systemic immune activation by RT (124, 125). A review by Pangal, et al, described multiple instances of patients with intracranial or intraspinal metastatic disease who had other distant tumors that had a response, despite no direct radiation therapy (126). Of note, most of these patients were on concurrent ICI therapy. This suggests that radiation could potentially “prime” the immune system, making ICI therapy more effective (127, 128). Another effect RT is thought to have relevant to CNS tumors is disruption of the BBB. Both preclinical and clinical studies have demonstrated radiation influences the integrity of the BBB (129–131). While this could put the CNS at increased risk of exposure to toxins and infection, it could also be advantageous. Disruption of the BBB could allow better penetration of systemic therapies and possibly improved immune infiltration, for better tumor control.

### 4.3 Overcoming immune evasion

Tumor cells have many ways to evade the immune system, and one is decreased expression of MHC-I (132, 133). MHC-I presents tumor peptides, which then are recognized by the T-cell receptor (TCR) of CD8+ T-cells. This in turn causes activation of the T-cell and induces tumor cell kill. Multiple studies have shown, even at low doses, RT can increase the expression of MHC-I on the surface. In work by Das, et al (134), exposure of human medulloblastoma cell lines to a low dose (1Gy) of ionization radiation increased the expression of MHC-I and MHC-II on the surface of these cells. Sharma’s, et al. (135) work showed similar findings: multiple cancer cell lines and normal human cells were exposed to a single dose of 20Gy radiation and showed increased expression of MHC-I and cancer testis antigen (CTA). This effect is advantageous because, in addition to the mechanisms already described, it is another way to overcome tumor immune evasion (136–138). Radiation has also been shown to increase the expression of other mAb targets. Wattenberg, et al. (139) demonstrated that exposing human cell lines to a single dose of either 5Gy or 10Gy of ionizing radiation significantly increased the expression of multiple targetable surface antigens (HER2, EGFR, and CD20). Similarly, Garnett, et al. (140) exposed human cell lines to low-dose radiation. The results showed an increase in the expression of Fas (CD95), in addition to other molecules that are involved in T-cell mediated cell tumor cell kill (such as intercellular adhesion molecule 1, mucin-1, carcinoembryonic antigen, and MHC I). In addition, some of the cell lines that did upregulate CEA showed enhanced CTL-mediated cell kill. In addition to antibody targets and MHC, radiation can increase immune cells directly. This has been demonstrated in multiple other pre-clinical studies (141–143). For example, Pandey, et al. (143) demonstrated in a preclinical model that a low dose of radiation increases immunogenicity by increasing the activation of CD8+ lymphocytes and increasing the phagocytic activity of macrophages. Therefore, we now know radiation affects both tumor cells and immune cells to further increase immunogenicity and enhance immunotherapies when used in combination.

### 4.4 Combination immune therapies

Due to the myriad effects radiation therapy has on the systemic immune system, there are multiple clinical trials to examine the effects of a combination of radiation therapy and various immune-oncologic treatments including ICIs, mAbs, and vaccines (137). Research has consistently shown that while immune strategies such as vaccinations or ICIs are not as effective on their own, there does seem to be better efficacy when these therapies are used in combination with each other or with other therapies (144–146). For example, ICIs in combination with vaccinations can overcome immune evasion and improve the efficacy of both therapies and increase CTL-mediated tumor cell kill (80, 146). The current landscape of immunotherapy for pediatric CNS tumors is heavily focused on complementary immunotherapies for better tumor control. **Table 3** outlines current open clinical trials for pediatric patients using combined immunotherapy modalities.

**Table 3:** Current open clinical trials for pediatric patients using combined immunotherapy modalities.

Clinical Trial Identifier	Drug	Population	Phase of Study	Status
NCT03396575	Focal RT, dose-intensified temozolomide, Total tumor mRNA-pulsed autologous Dendritic Cells (TTRNA-DCs), Tumor-specific ex vivo expanded autologous lymphocyte transfer (TTRNA-xALT) Autologous hematopoietic stem cells (Auto-HSC)	Patients aged 3-30 years with radiologically confirmed DIPG or other diffuse intrinsic brain stem glioma (Grade III or IV)	I	Active, recruiting
NCT05457959	Dendritic cell tumor peptide vaccine with ipilimumab and nivolumab after resection	Patients aged 13-60 years with recurrent/progressive diffuse hemispheric glioma, H3G34-Mutant	I	Not yet recruiting
NCT04943848	rHSC-DIPGVax with balstilimab and zalifrelimab	Patients aged 12 months-18 years with newly diagnosed typical or non-typical, biopsy-proven DIPG or DMG who have received standard-of-care radiation therapy	I	Active, recruiting
NCT02960230	H3.3.K27M epitope synthetic peptide vaccine with poly-ICLC +/- nivolumab	Patients aged 3-21 who underwent standard radiation therapy with newly diagnosed DIPG who are positive for the H3.3K27M mutation (stratum A), newly diagnosed glioma other than DIPG who are positive for the H3.3K27M mutation including spinal cord gliomas (stratum B), newly diagnosed DIPG or midline glioma other than DIPG (excluding primary spinal cord gliomas) who are positive for the H3.3K27M mutation (stratum C)	I/II	Active, not recruiting
NCT03690869	Cemiplimab with radiation therapy	Patients aged 0-18 years (Phase 1) or 3-25 years (Efficacy phase) with newly diagnosed diffuse intrinsic pontine glioma (DIPG), HGG, or recurrent HGG	I/II	Active, recruiting
NCT04911621	Autologous Wilms’ tumor-1 (WT1) mRNA-loaded dendritic cell vaccine, with chemoradiotherapy with temozolomide +/- chemoimmunotherapy with temozolomide	Patients aged 12 months to 18 years with histologically verified HGG (WHO grade III or IV) or radiologically confirmed DIPG who are newly diagnosed (stratum A) or have received prior therapy (stratum B)	I/II	Active, not recruiting

## 5 Immunotherapy-associated toxicities

As with all therapies, the risk-to-benefit ratio is an important consideration when choosing therapies for extremely young patients with developing nervous systems. The side effects of immunotherapies are becoming more well-known. These off-target effects (specifically neurologic immune-related adverse events, (NirAEs)) are estimated to occur anywhere from 1-6% (147–150) and can be both local (tumor edema) and distant immune cell-mediated. These NirAEs include issues such as myositis, myasthenia symptoms, peripheral and cranial neuropathies, encephalopathy, and seizures (151–154). As discussed earlier, pseudoprogression is a not infrequent occurrence as well which can confuse the evaluation of disease response (however, data suggests that the incidence of pseudoprogression in some patients is related to better response to immunotherapy).

Treatment of such neurotoxicities usually includes holding the immunotherapy until symptoms improve and treating neurologic issues in standard fashion (for example, anti-epileptic medications for seizures or plasmapheresis for Guillain-Barré syndrome) (152). An additional mainstay of therapy is corticosteroids (150, 154). Other immunomodulators have also been suggested, especially in cases that are refractory to steroids or can be life-threatening (153, 154).

## 6 Conclusion

CNS tumors are the number one cause of cancer deaths in pediatric patients. Multiple modalities of therapy have shown varying degrees of efficacy in pediatric patients. The CNS immune system is very complicated, and with our ever-increasing knowledge of it, along with sophisticated and detailed molecular diagnostic techniques, the ability to target the unique features of the CNS immune system is much more in reach. Combination therapies, new ways of delivery of vaccines, and promising preclinical data for even more antibody-mediated modes of therapy are indications of the potential to bring these therapies to pediatric patients and increase the survival of some of the most devastating childhood diseases.

## Author contributions

The author confirms sole responsibility for the following: article conception and design, data collection, analysis, and manuscript preparation.
